# New insights into the function of mammalian Argonaute2

**DOI:** 10.1371/journal.pgen.1009058

**Published:** 2020-11-12

**Authors:** Marek Marzec

**Affiliations:** University of Silesia in Katowice, Faculty of Natural Sciences, Institute of Biology, Biotechnology and Environmental Protection, Katowice, Poland; University of California Los Angeles, UNITED STATES

## Abstract

Uncovering the mechanisms that recognise a microRNA (miRNA) target is 1 of the biggest challenges because the Ago–miRNA complex is able to overcome different derogations of complementarity when binding targets. However, the recently solved crystallographic structure of Argonaute2 (Ago2) and a high-throughput analysis that used repurposed sequencing techniques has brought us closer to achieving this goal.

MicroRNAs (miRNAs) are a group of short (approximately 22 nucleotides) noncoding RNAs that are used by the Argonaute (Ago) proteins as guides for identifying complementary sites in the messenger RNAs (mRNAs) that are targeted for repression [1]. It has been estimated that the expression of more than 50% of the protein-coding genes in humans are under miRNA control [2]. miRNAs are categorised into families that are based on the sequence similarity, and each miRNA is able to target multiple mRNAs. The most conserved part of miRNA is located at 5′-end and is called the seed region. Guide (g) nucleotides from the seed region (g2–g7 or g2–g8) play a primary role in target recognition because more than 80% of the interactions between miRNAs and targets occurs via seed pairing [3]. However, for 20% of the miRNA–target interactions, other regions such as the supplementary region (g13–g16) of miRNA might be involved in targeting [4,5]. Although the miRNAs that belong to the same family share the same seed sequence, they contain divergent 3′ regions. A functional redundancy has been described for representatives of the same family; however, there are also some examples in which a single miRNA recognises a specific gene [6].

Argonaute2 (Ago2) contains 3 RNA-binding domains: a P-element-induced whimpy testes (PIWI) domain, a middle (MID) domain, and a PIWI/Argonaute/Zwille (PAZ) domain. These RNA-binding domains are responsible for distinct tasks in binding of mature miRNAs: MID domain is responsible for anchoring the mono-phosphorylated 5′-end, and PAZ domain binds the hydroxylated 3′-end [7,8]. Current structural insights into Ago-mediated miRNA targeting in animals indicate that Ago creates a “seed chamber” for efficient and stable association with seed-paired target RNAs [8]. In contrast to the previous work which focused on the crystal structure of seed chamber, Sheu-Gruttadauria and colleagues described the crystal structure of the human Ago2 protein, which is where the base pairing of both the seed and the supplementary regions between miRNA and the target occurs [9]. In that situation, Ago2 opens an additional chamber (supplementary chamber) for up to 4 additional base pairs of the miRNA–target duplex (Fig 1). It was proven that the seed- and supplementary-paired regions in an mRNA sequence might be separated by 1 to 15 nucleotides, which increased the target affinity by more than 10-fold (when a guanine/cytosine (GC)-rich supplementary sequence was used) compared to seed-only pairing. The largest increase in affinity (more than 20-fold) was observed when 2 to 4 nucleotides created a bridge between a seed and the supplementary sequence in mRNA. Adenine/uracil (AU)-rich sequences had a weaker effect on affinity; however, for a 4-nucleotide bridge, there was a 10-fold increase. Moreover, an analysis of the miRNA isoforms that contain additional 3′ nucleotides (3′ isomiRs) revealed that longer 23-nucleotide 3′ isomiRs stabilise the miRNA–target interaction compared to the 21-nucleotide 3′ isomiRs. Additionally, it was proven that Ago2 can use supplementary interactions without any central (g9–g12) pairing, and this situation is energetically favoured for miRNA–target pairing beyond the seed [9]. Moreover, it should be stressed that supplementary pairing plays various roles in increasing the target affinity for different miRNAs, which was also proven by Sheu-Gruttadauria and colleagues. Because the seed region can be separated by up to 15 nucleotides from supplementary region, and only 2 GC supplementary base pairs can significantly increase target affinity, it cannot be excluded that a lot of supplementary sites were overlooked in previous studies. According to the miRBase, about 25% of human miRNAs contain ≥3 GC nucleotides within positions g13–g16, which pointed out how important is the findings of Sheu-Gruttadauria and colleagues [9]. Similar results were obtained by 2 other research groups that used a high-throughput analysis to uncover the rules of miRNA target recognition. Becker and colleagues described the methods to measure the binding affinity and cleavage rates that was examined for mouse Ago2 that had been loaded with 2 different miRNAs (let-7a (seed-only pairing) and miR-21 (seed and supplementary pairing)) [10]. An Ilumina MiSeq platform was used to create a library of approximately 20,000 distinct RNA targets per miRNA including targets with various mismatches or indels as well as targets that had been predicted by different algorithms. Binding affinity was measured using a catalytically inactive Ago2 mutant (D669A) that had been loaded with fluorescent-labelled miRNA, and the wild-type Ago2 was used to measure the cleavage rates. The results confirmed that a miRNA–target complementarity in the seed region plays a crucial role in binding affinity. Similarly, when indels were present in the target sequence complementary to the miRNA seed, the binding affinity of miRNA to the target RNA was reduced. However, deletions had a stronger effect than insertions at the same target positions. For some miRNAs with a low GC content in the seed region (such as miR-21), mismatches in the 3′ supplementary region have a similar effect in reducing affinity as those in the seed region. In that case, pairing in the supplementary region is necessary for a high-affinity binding. It was also proven that insertions in the target sequence, which is opposite the central region of the miRNA, do not affect binding affinity [10]. This is in agreement with the findings of Sheu-Gruttadauria and colleagues [9], according to which the seed- and supplementary-paired regions in an mRNA sequence might be separated by up to 15 nucleotides. Additionally, binding to the noncanonical target sequences (that contain less than 6 contiguous nucleotide matches to the seed region of a miRNA) was analysed. For the majority of the analysed noncanonical targets that had been predicted by different algorithms, no interactions with miRNA were detected (95% for miR-21 and 89% for let-7a) [10]. However, it might be that the predictions of noncanonical sites by different algorithms were incorrect. This explanation is supported by the work of McGeary and colleagues [11]. It was possible to analyse the binding affinity of miRNAs to RNA random sequences using an RNA Bind-n-Seq (RBNS). This high-throughput approach enables all known canonical sites to be analysed and new noncanonical sites for 6 different miRNAs (miR-1, let-7a, miR-7, miR-124, miR-155, and lsy-6) to be described. For example, after an analysis of 1,398,016 motifs up to 10 nucleotides long, 8 previously unknown sites with a binding affinity similar to 6 nucleotide seed sequences were identified for miR-1. Moreover, for other miRNAs, the new noncanonical sites were also characterised. The results not only clearly indicated differences in the noncanonical site types observed for the analysed miRNAs but also miRNA-specific differences in the affinities of the canonical sites. However, 1 observation was highly consistent between the different miRNAs and miRNA site types: the dinucleotides that flanked the canonical sequence had a great impact (up to 100-fold) on the binding affinity. An analysis of 6 miRNAs revealed that A/U enhanced affinity, G reduced affinity, and C did not affect affinity. This effect was highly consistent between the different miRNAs and miRNA site types. The results can be explained by the impact of the nucleotides on the stabilisation of the RNA secondary structure; a lower propensity to stabilise the secondary structure results in reduced changes to abolishes binding [10].

**Fig 1:**
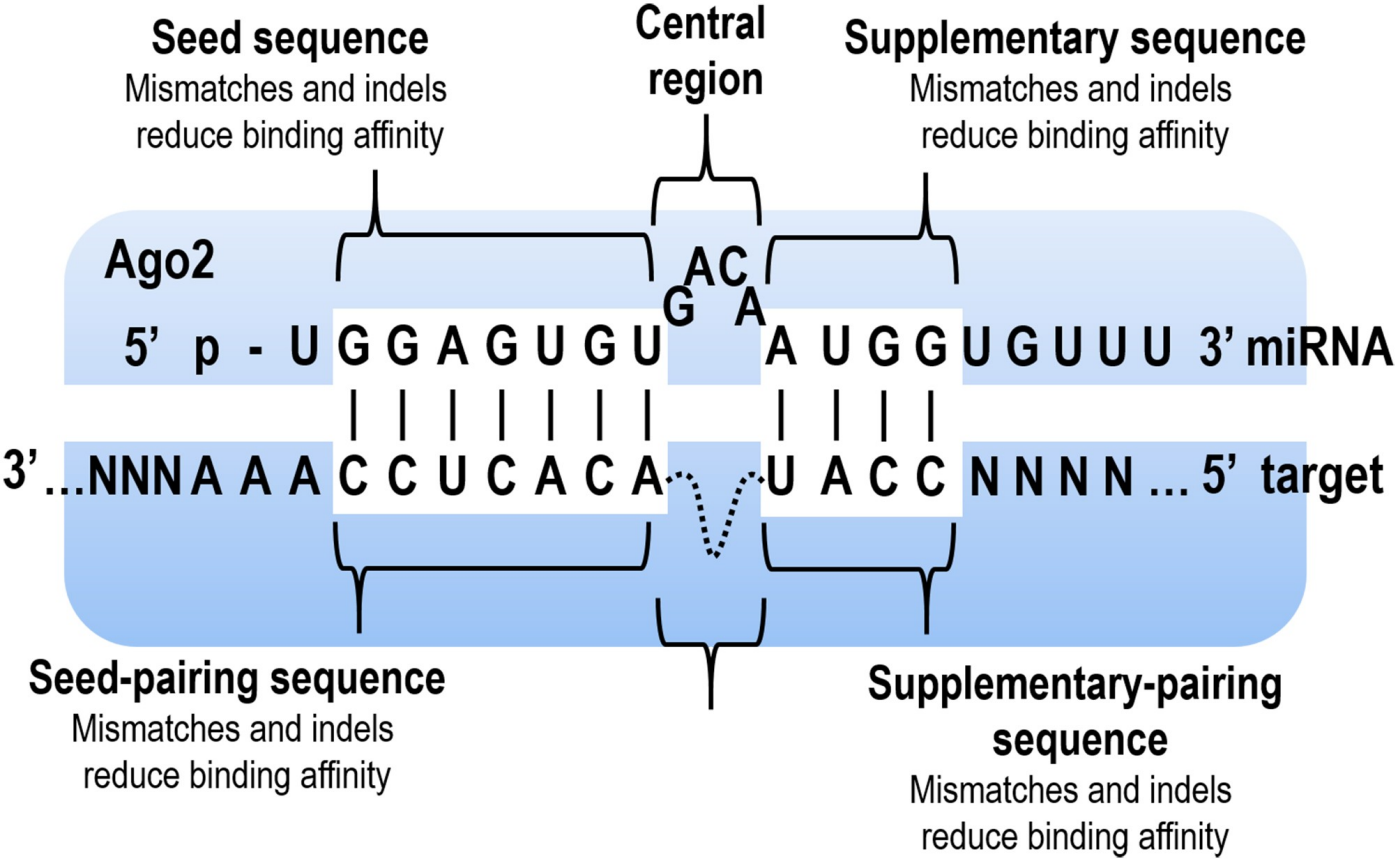
Overview of the miRNA–target interactions in the human Ago2. Ago, Argonaute2; miRNA, microRNA.

Last year saw great progress in uncovering the mechanisms that are related to miRNA targeting. First of all, different conformations of the human Ago2 have been described, and secondly, high-throughput analysis, which is used to measure the binding affinities of different RNAs to miRNAs, enables the biochemical basis of miRNA targeting to be described. When the results that had been obtained in only 1 of the described works were used to train a convolutional neural network, the prediction of the miRNA repression outperformed all of the previously used algorithms. Using other results, i.e., information that 1 to 15 nucleotides can separate the seed- and supplementary-pairing regions, will enable the further improvement of target prediction algorithms. Using of the methods that have already described and adapting the new high-throughput methods will undoubtedly provide further insights into miRNA targeting. Also, new classes of miRNA were recently described, i.e., class of miRNA target sites that lack both perfect seed pairing and 3′-compensatory pairing and instead have 11 to 12 contiguous Watson–Crick pairs to the center of the miRNA [12] or class of miRNA recognition elements that function exclusively in coding sequence (CDS) regions [13]. Now, 1 of the biggest challenge will be to find a way for identification of supplementary sites that can significantly increase target affinity and include them in the target prediction algorithms.
